# Dataset of phytoplankton productive parameters and environmental forces in autumn in the Kara Sea

**DOI:** 10.1016/j.dib.2019.01.001

**Published:** 2019-01-06

**Authors:** Sergey A. Mosharov, Valentina M. Sergeeva, Vyacheslav V. Kremenetskiy, Andrey F. Sazhin, Svetlana V. Stepanova

**Affiliations:** Shirshov Institute of Oceanology, Russian Academy of Sciences, Nakhimovskiy prospect 36, 117997 Moscow, Russia

## Abstract

This data article contains data on the photosynthetic activity, shares of the main algae groups in the total phytoplankton biomass and the main environmental forces in the Kara Sea. The data were collected from 17 to 29 of September, 2011 in the following different areas: the Kara Sea Shelf with and without river runoff influence, the shelf edge and continental slope. The photosynthetic activity parameters include the relative electron transport rate (rETR) values, the maximum quantum efficiency of PSII (Fv/Fm) and the P^B^/rETR ratio as photosynthetic performance. The main environmental forces include the average day incident light, salinity and water temperature in the upper mixed layer and dissolved nutrients. The data presented in this article are associated with the research article entitled “Assessment of phytoplankton photosynthetic efficiency based on measurement of fluorescence parameters and radiocarbon uptake in the Kara Sea” (Mosharov et al., 2019). The related research article examines the relationships between biological parameters and with environmental characteristics such as temperature, salinity, incident photosynthetically active radiation (PAR) and nutrient concentration.

**Specifications table**

**Table:** 

Subject area	Biology
More specific subject area	Phytoplankton ecology
Type of data	Table
How data were acquired	PAM-Fluorometer (Mega-25); the 14C uptake method and ICES photosynthetron (Hydro-Bios), Li-190 Quantum Sensor and Li-1400 data logger (Li-Cor); Microscope (Leica DM 500B); CTD (SBE-19 Plus; Sea Bird Equipment)
Data format	Raw, filtered, analysed
Experimental factors	Prior to fluorescence measurements, the water samples were kept in the dark for 20 min. Prior to microscopy counts of phytoplankton cells, water samples were concentrated via inverse filtration (large cells) or stained with fluorochrome primulin, fixed with 3.6% glutaraldehyde solution and filtered through black 0.4-μm pore-size Nuclepore filters (for small cells).
Experimental features	Productive activity of phytoplankton was measured experimentally with two different techniques: fluorescence and carbon fixation estimations.
Data source location	The Kara Sea; 24 stations between 73°43′–78°28′ N and 69°59′–86°39′ E
Data accessibility	Relevant data reported in this article
Related research article	S. А. Mosharov, V. М. Sergeeva, V. V. Kremenetskiy, А. F. Sazhin, S.V. Stepanova. Assessment of phytoplankton photosynthetic efficiency based on measurement of fluorescence parameters and radiocarbon uptake in the Kara Sea. Estuarine, Coastal and Shelf Science (2019) [1]

**Value of the data**

• The dataset can be used to study the variability in phytoplankton fluorescence parameters from samples collected in the polar region, where there is low solar radiation.
• Samples were collected along the Kara Sea Shelf with and without river runoff influence, the continental shelf edge and the continental slope. Consequently, these data can be used as a reference for the analysis of ecological variability in coastal and shelf areas.
• The dataset can be used for comparative analysis of light and dark processes of phytoplankton photosynthesis in the different nature conditions.

## 1 Data

The data presented in this article are related to the estimation of the variability of photosynthetic activity of phytoplankton communities from the surface waters of the Kara Sea. Data are present for the following regions in the Kara Sea: the Kara sea shelf with Yenisei river runoff influence, the eastern coastal shelf, the central shelf near St. Anna trough, the edge of continental shelf and the slope of the St. Anna trough [1]. The sampling was carried out on the research vessel *Akademic Mstislav Keldysh* in September, 2011. The areas were divided based on the direct measurements of hydrophysical and nutrient data (Table 1). One notably important feature of the data set is that simultaneous measurements of all above mentioned biological and environmental parameters were conducted.

**Table 1:** Abiotic parameters for different regions in surface waters.

Area	Date	Long	Lat	Depth	PAR/day	Sal UML	T UML	DIN	DIP	Si
Yenisei SHELF	20 Sept	79.4	73.7	32	52	21.9	5.0	1.9	0.44	29.2
Yenisei SHELF	21 Sept	78.9	74.0	29	85	17.8	5.0	1.4	0.38	24.8
Yenisei SHELF	22.Sept	77.9	75.0	39	220	24.6	5.2	0.7	0.16	14.9
Yenisei SHELF	22 Sept	77.2	75.6	48	143	23.0	5.4	1.0	0.12	18.1
Yenisei SHELF	17 Sept	78.6	74.3	33	100	26.4	4.8	0.6	0.2	8.2
Eastern SHELF	24 Sept	80.8	76.6	59	82.65	28.7	4.1	0.4	0.3	3.8
Eastern SHELF	23 Sept	85.4	75.4	55	80.6	29.2	3.8	1.2	0.21	2.9
Eastern SHELF	23 Sept	86.7	75.2	40	80.6	18.9	3.9	2.1	0.33	29.0
Eastern SHELF	23 Sept	85.6	75.3	39	80.6	24.4	3.0	1.5	0.34	17.8
St. Anna SHELF	28 Sept	72.6	76.3	140	50	19.0	5.5	0.9	0.18	30.7
St. Anna SHELF	25 Sept	78.1	77.2	125	87.75	27.4	4.7	0.8	0.12	8.2
St. Anna SHELF	22 Sept	76.7	76.0	64	125.4	24.3	5.7	0.6	0.17	17.3
EDGE of St. Anna SHELF	28 Sept	71.7	76.5	158	50	24.1	4.1	1.34	0.17	4.3
EDGE of St. Anna SHELF	29 Sept	71.5	76.6	181	56	27.0	4.2	1.0	0.15	14.4
EDGE of St. Anna SHELF	29 Sept	71.2	76.6	244	56	26.6	4.7	1.1	0.14	8.6
EDGE of St. Anna SHELF	29 Sept	71.0	76.7	320	31.36	29.6	4.1	1.23	0.12	7.83
EDGE of St. Anna SHELF	25 Sept	77.7	77.4	196	117	27.4	4.7	0.81	0.12	7.59
EDGE of St. Anna SHELF	25 Sept	77.5	77.4	219	91.26	31.2	3.9	1.37	0.12	1.76
St. Anna SLOPE	29 Sept	70.5	76.8	492	22.4	34	3.5	0.78	0.05	1.04
St. Anna SLOPE	29 Sept	70.0	77.0	536	56	33.5	3.6	0.71	0.05	1.09
St. Anna SLOPE	25 Sept	76.0	77.8	321	117	31.6	3.4	1.49	0.09	0.74
St. Anna SLOPE	26 Sept	74.8	78.0	364	51	31.6	3.4	1.04	0.11	0.26
St. Anna SLOPE	26 Sept	73.6	78.3	414	20.4	32.8	3.4	0.6	0.06	0.14
St. Anna SLOPE	26 Sept	72.8	78.5	472	51	32.1	2.9	0.42	0.04	0.12

Long – longitude (°N); Lat – latitude (°E); Depth – total depth (m); PAR/day - average-day incident PAR (μmol photons m^−2^ s^−1^); S UML – salinity of upper mixed layer (psu); T UML – temperature of upper mixed layer (°C); DIN – dissolved inorganic nitrogen (DIN = NO_2_ + NO_3_ + NH_4_, μM), DIP – dissolved inorganic phosphorus (μM), Si – dissolved inorganic silicon (μM).

Two different techniques were used to determine the physiological activity of phytoplankton: fluorescence measurements and experimental carbon fixation estimations. The productive potential of phytoplankton was characterized by follow parameters: the relative electron transport rate (rETR_0_) values, the maximum quantum efficiency of photosystem II (PSII) (F_v_/F_m_), photosynthetic performance (P^B^_0_/rETR_0_ ratio, where P^B^_0_ is the biomass-specific primary production [2]) and chlorophyll *a* concentration. Those variables that are characteristic of phytoplankton structure were presented as shares of the main groups in the total phytoplankton biomass. These groups include dinoflagellates, diatoms, spores of diatoms, autotrophic flagellates and heterotrophs (Table 2).

**Table 2:** Biological parameters for different regions in surface waters.

Area	Date	Long	Lat	Fv/Fm	rETR_0_	PB_0_/rETR_0_	Chl *a*	% DINO	% DIA	%DIA spore	% FLA	% HET
Yenisei SHELF	20 Sept	79.4	73.7	0.706	14	0.02	1.093	0.4	25.7	1.4	22.3	50.3
Yenisei SHELF	21 Sept	78.9	74.0	0.665	22	0.03	1.067	8.8	27	5.9	30.4	27.8
Yenisei SHELF	22.Sept	77.9	75.0	0.652	28.3	0.04	0.457	10.7	62.1	1.6	16.8	8.9
Yenisei SHELF	22 Sept	77.2	75.6	0.639	38	0.05	1.047	6.9	60.4	2.2	8.1	22.4
Yenisei SHELF	17 Sept	78.6	74.3	0.32	19.3	0.00	0.349	0.04	66.9	1.3	11.8	19.9
Eastern SHELF	24 Sept	80.8	76.6	0.466	17.4	0.07	0.272	34.8	14.9	23.3	8.8	18.1
Eastern SHELF	23 Sept	85.4	75.4	0.624	25.8	0.03	0.204	n/a	n/a	n/a	n/a	n/a
Eastern SHELF	23 Sept	86.7	75.2	0.666	30.88	0.04	0.775	8.9	72.9	0.3	4.9	13
Eastern SHELF	23 Sept	85.6	75.3	0.578	21.47	0.04	0.405	71.5	12.3	4.9	7.2	4.1
St. Anna SHELF	28 Sept	72.6	76.3	0.594	12.13	0.04	1.493	3.4	0.3	0.01	45.2	51.2
St. Anna SHELF	25 Sept	78.1	77.2	0.6	24	0.04	0.682	22.5	33.2	0.8	22.3	21.3
St. Anna SHELF	22 Sept	76.7	76.0	0.626	29.6	0.05	0.806	34.6	4.6	0	14.3	46.5
EDGE of St. Anna SHELF	28 Sept	71.7	76.5	0.645	11.46	0.02	2.289	50.7	8.3	0	25.1	15.9
EDGE of St. Anna SHELF	29 Sept	71.5	76.6	0.581	11.49	0.02	1.35	25.7	1.6	0.4	36.7	35.6
EDGE of St. Anna SHELF	29 Sept	71.2	76.6	0.604	10.94	0.02	0.754	9.1	7.7	11.4	23.1	48.6
EDGE of St. Anna SHELF	29 Sept	71.0	76.7	0.629	13.67	0.01	1.139	39.4	3.8	0.1	34.3	22.3
EDGE of St. Anna SHELF	25 Sept	77.7	77.4	0.611	22.17	0.03	1.16	58.7	30.8	0.1	0.1	7.3
EDGE of St. Anna SHELF	25 Sept	77.5	77.4	0.559	24.83	0.04	0.621	25.1	15.8	0.7	36.2	22.1
St. Anna SLOPE	29 Sept	70.5	76.8	0.55	7.35	0.04	0.554	6.1	0.1	0	61.4	32.5
St. Anna SLOPE	29 Sept	70.0	77.0	0.638	9.67	0.02	0.457	19.4	0.2	0.5	41.5	38.5
St. Anna SLOPE	25 Sept	76.0	77.8	0.714	25.21	0.02	0.462	28.4	5.3	0.5	36.5	29.3
St. Anna SLOPE	26 Sept	74.8	78.0	0.647	12	0.03	0.431	41.4	1.3	1.7	23.9	31.8
St. Anna SLOPE	26 Sept	73.6	78.3	0.608	5.58	0.05	0.554	16.9	5.5	8.9	23.5	45.2
St. Anna SLOPE	26 Sept	72.8	78.5	0.564	4.87	0.03	0.472	22.4	24.3	1.1	23.2	28.9

Long - longitude (°N); Lat – latitude (°E); F_v_/F_m_ – PSII efficiency; rETR_0_ – relative electron transport rate; P^B^_0_ /rETR_0_ – ratio between the biomass-specific primary production and relative electron transport rate; Chl *a* –chlorophyll *a* concentration (mg m^-3^); share of different algae groups in the total phytoplankton biomass (%): %DINO – autotrophic dinoflagellates, %DIA – active diatoms, %DIAspore – diatom spores, %FLA – flagellates, %HET – heterotrophs.

## 2 Experimental design, materials and methods

### 2.1 Sampling

Surface water samples were collected from 24 stations (between 73 °43′–78 °28′ N and 69 °59′–86 °39′ E) in the Kara Sea. The coordinates of the sampling sites are in Tables 1 and 2. The water samples were divided into subsamples, which were used for measurement of different parameters like nutrient and chlorophyll concentrations, experimental carbon fixation estimations (primary production), chlorophyll fluorescence, and abundance and biomass of phytoplankton.

### 2.2 Phytoplankton productivity parameters measurement

Primary production (PP) was measured onboard using the ^14^C uptake method [3] and exposed in the photosynthetron (Hydro-Bios, Germany). Biomass-specific PP, P^B^ (mg C (mg chlorophyll *a*)^−1^ day^−1^) was calculated by normalizing PP at different depths to the corresponding chlorophyll *a* concentration. The chlorophyll *a* concentration was measured fluorometrically [4] after filtering onto Whatman GF/F (glass-fibre filters). Surface irradiance (400–700 nm) was measured with a Li-Cor Li-190 Quantum Sensor. Active chlorophyll *a* fluorescence (Fv/Fm) was measured with a MEGA-25 PAM-fluorometer (MSU, Russia) after 30 minutes of dark adaptation. Water samples were exposed in the PAM fluorometer to eight light intensities as for the ^14^C uptake measurements, for 300 s at each step, and steady fluorescence (*F*_t_) and maximum fluorescence (*F*_m_′) were measured and the resultant rETR values were estimated.

### 2.3 Determination of shares of different phytoplankton groups

Phytoplankton cell counts were carried out using luminescent microscopy for small phytoplankton forms with linear size <10–15 µm on black 0.4 µm pore-size Nuclepore filters [5]. Larger phytoplankton was counted using the concentrate obtained by reversed filtration method [6]. Determination of phytoplankton composition, abundance and biomass were performed with a Leica DM2 500 light microscope. The algae species constructive metabolism type was determined according to the literature [7], [8]. Published allometric dependences were used to convert wet phytoplankton biomass to carbon units [9].
