# Supplementary material for: Dataset of phytoplankton productive parameters and environmental forces in autumn in the Kara Sea
Source: Data Brief. 2019 Jan 6;22:821–5. doi: 10.1016/j.dib.2019.01.001 (PMC6348287; doi:10.1016/j.dib.2019.01.001)
Supplement: Supplementary file 1 — Supplementary material [file mmc1.docx]

# Conflict of interest:

We wish to confirm that there are no known conflicts of interest associated with this publication and there has been no significant financial support for this work that could have influenced its outcome.

We confirm that the manuscript has been read and approved by all named authors and that there are no other persons who satisfied the criteria for authorship but are not listed. We further confirm that the order of authors listed in the manuscript has been approved by all of us.

S. А. Mosharov,

V. М. Sergeeva,

V. V. Kremenetskiy,

А. F. Sahzin,

С. В. Stepanova
